# Supplementary material for: Natural Polyphenol-Containing Gels against HSV-1 Infection: A Comparative Study
Source: Nanomaterials (Basel). 2022 Jan 11;12(2):227. doi: 10.3390/nano12020227 (PMC8780422; doi:10.3390/nano12020227)
Supplement: Supplementary file 1 [file nanomaterials-12-00227-s001.zip › nanomaterials-1532162-supplementary.pdf]

Supplementary material

Natural Polyphenol-Containing Gels against HSV-1 Infection: A Comparative Study

Mariaconcetta Sicurella [1], Maddalena Sguizzato [1], Paolo Mariani [2], Alessia Pepe [2], Anna Baldisserotto [3], Raissa Buzzi [3], Nicolas Huang [4], Fanny Simelière [4], Sam Burholt [5], Peggy Marconi [1,*] and Elisabetta Esposito [1,*]

[1] Department of Chemical, Pharmaceutical and Agricultural Sciences, University of Ferrara, I-44121 Ferrara, Italy; scrmcn@unife.it (M.S.); sgzmdl@unife.it (M.S.)

[2] Department of Life and Environmental Sciences, Università Politecnica delle Marche, I-60131 Ancona, Italy;
p.mariani@staff.univpm.it (P.M.); a.pepe@pm.univpm.it (A.P.)

[3] Department of Life Sciences and Biotechnology, University of Ferrara, I-44121 Ferrara, Italy; anna.baldisserotto@unife.it (A.B.); raissa.buzzi@unife.it (R.B.)

[4] CNRS, Institut Galien Paris-Saclay, Université Paris-Saclay, 92296 Châtenay-Malabry, France; nicolas.huang@universite-paris-saclay.fr (N.H.); fanny.simeliere@universite-paris-saclay.fr (F.S.)

[5] Diamond Light Source Ltd., Harwell Science and Innovation Campus, Didcot OX11 0DE, Oxfordshire, UK; sam.burholt@diamond.ac.uk

* Correspondence: mcy@unife.it (P.M.); ese@unife.it (E.E.); Tel.: +39-0532-455230 (E.E.)

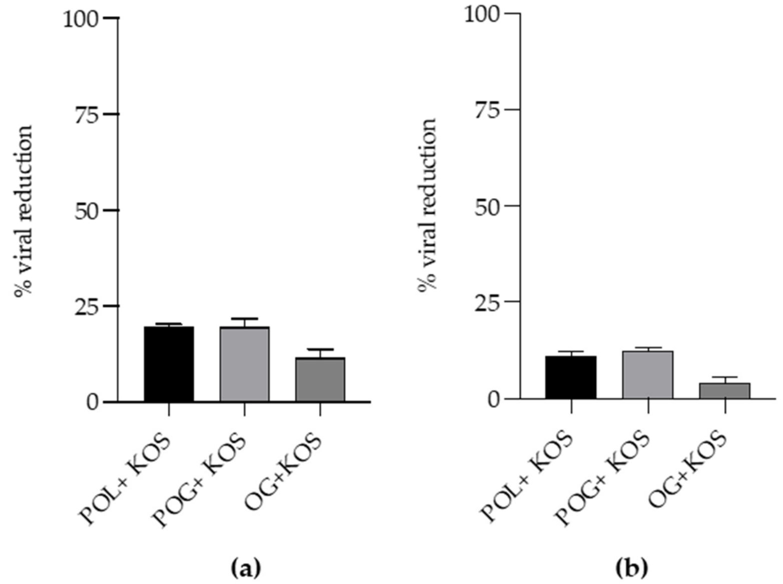

Figure S1. Virucidal activity of unloaded gels against HSV-1 strain KOS at 35 °C, expressed as percentage of viral reduction measured after 1 h (**a**) and 6 h (**b**) of direct contact.
